# The Impact of Trachomatous Trichiasis on Quality of Life: A Case Control Study

**DOI:** 10.1371/journal.pntd.0004254

**Published:** 2015-11-23

**Authors:** Esmael Habtamu, Tariku Wondie, Sintayehu Aweke, Zerihun Tadesse, Mulat Zerihun, Zebideru Zewudie, Wondimu Gebeyehu, Kelly Callahan, Paul M. Emerson, Hannah Kuper, Robin L. Bailey, David C. W. Mabey, Saul N. Rajak, Sarah Polack, Helen A. Weiss, Matthew J. Burton

**Affiliations:** 1 International Centre for Eye Health, London School of Hygiene & Tropical Medicine, London, United Kingdom; 2 The Carter Center, Addis Ababa, Ethiopia; 3 Amhara Regional Health Bureau, Bahirdar, Ethiopia; 4 The Carter Center, Atlanta, Georgia, United States of America; 5 International Trachoma Initiative, Atlanta, Georgia, United States of America; 6 International Centre for Evidence in Disability, London School of Hygiene & Tropical Medicine, London, United Kingdom; 7 Clinical Research Department, London School of Hygiene & Tropical Medicine, London, United Kingdom; 8 Department of Infectious Disease Epidemiology, London School of Hygiene & Tropical Medicine, London, United Kingdom; RTI International, UNITED REPUBLIC OF TANZANIA

## Abstract

**Background:**

Trachomatous trichiasis is thought to have a profound effect on quality of life (QoL), however, there is little research in this area. We measured vision and health-related QoL in a case-control study in Amhara Region, Ethiopia.

**Methodology/Principal Findings:**

We recruited 1000 adult trichiasis cases and 200 trichiasis-free controls, matched to every fifth trichiasis case on age (+/- two years), sex and location. Vision-related quality of life (VRQoL) and health-related quality of life (HRQoL) were measured using the WHO/PBD-VF20 and WHOQOL-BREF questionnaires. Comparisons were made using linear regression adjusted for age, sex and socioeconomic status. Trichiasis cases had substantially lower VRQoL than controls on all subscales (overall eyesight, visual symptom, general functioning and psychosocial, p<0.0001), even in the sub-group with normal vision (p<0.0001). Lower VRQoL scores in cases were associated with longer trichiasis duration, central corneal opacity, visual impairment and poor contrast sensitivity. Trichiasis cases had lower HRQoL in all domains (Physical-health, Psychological, Social, Environment, p<0.0001), lower overall QoL (mean, 34.5 v 64.6; p<0.0001) and overall health satisfaction (mean, 38.2 v 71.7; p<0.0001). This association persisted in a sub-group analysis of cases and controls with normal vision. Not having a marriage partner (p<0.0001), visual impairment (p = 0.0068), daily labouring (p<0.0001), presence of other health problems (p = 0.0018) and low self-rated wealth (p<0.0001) were independently associated with lower overall QoL scores in cases. Among cases, trichiasis caused 596 (59%) to feel embarrassed, 913 (91.3%) to worry they may lose their remaining eyesight and 681 (68.1%) to have sleep disturbance.

**Conclusions/Significance:**

Trachomatous trichiasis substantially reduces vision and health related QoL and is disabling, even without visual impairment. Prompt trichiasis intervention is needed both to prevent vision loss and to alleviate physical and psychological suffering, social exclusion and improve overall well-being. Implementation of the full SAFE strategy is needed to prevent the development of trachomatous trichiasis.

## Author Summary

There is clear evidence that visual impairment generally reduces quality of life. However, relatively little is known about the impact that trachomatous trichiasis (TT) has on the lives of affected people with and without the presence of visual impairment. We measured the impact of TT on vision and health-related quality of life in 1000 people with TT using standard WHO quantitative tools and compared these with 200 trichiasis-free controls, matched to every fifth trichiasis case on age, sex and location. We found TT cases had lower vision and health related quality of life than controls regardless of visual impairment and other health problems suggesting the burden of TT goes beyond visual loss. The results provide solid data for advocacy and encourage programme leaders and funders to secure resources to promote trichiasis intervention. Trichiasis causes considerable physical and psychosocial trauma including sleep disturbance, low self-esteem and possibly a less stable marriage regardless of visual impairment. These suggest that, timely treatment is needed not only to prevent visual loss but also alleviate physical and psychological suffering and social exclusion of TT patients, thereby improving their physical and psychological health, general functioning and social relations.

## Introduction

Trachoma is the leading infectious cause of blindness worldwide [1]. About 229 million people live in trachoma endemic areas [2]. The disease starts in early childhood with repeated episodes of *Chlamydia trachomatis* infection. This triggers conjunctival inflammation of the upper eyelid, which leads to scarring. The scarring causes the eyelid to turn in (entropion) and the eyelashes to scratch the eye, which is known as trachomatous trichiasis (TT). Visual impairment and blindness develop when the cornea is damaged directly or indirectly by the trichiasis and ocular surface dysfunction, leading to corneal opacification (CO). Approximately 7.3 million people have un-treated trichiasis [3]. It is estimated that 2.4 million people are visually impaired from trachoma worldwide, among which between 439,000 and 1.2 million are estimated to be irreversibly blind [2,4].

Clinical examination provides little insight into the impact a condition has on the overall functioning and life of an affected individual and their family. Trichiasis can cause ocular pain and impaired vision but it can also have a profound effect on broader aspects of general health and well-being [5]. However, there is very limited data on the effect of trichiasis and its associated visual impairment on quality of life (QoL). QoL is a broad concept that refers to an “individual’s perceptions of their position in life in the context of the culture and value systems in which they live and in relation to their goals, expectations, standards and concerns” [6]. It can be measured quantitatively by a variety of tools including health-related quality of life (HRQoL) tools and tools measuring broader concepts to evaluate the overall experience of life [7]. HRQoL tools can be further divided into those measuring disease-specific quality of life (e.g. vision related QoL) and generic HRQoL [7].

A comprehensive vision related quality of life (VRQoL) measure has been developed by the World Health Organization (WHO): WHO/PBD-VF20 (World Health Organisation/ Prevention of Blindness and Deafness—Visual Functioning 20 item questionnaire) [8]. This tool was designed to explore the eyesight, ocular pain and discomfort, general functioning and psychosocial factors related to vision. WHO/PBD-VF20 has been evaluated and showed good psychometric properties in studies of people with visual impairment from cataract in Kenya, Bangladesh and the Philippines [9–11]. However, it has not been used to measure VRQoL in people living with trichiasis.

Generic HRQoL tools assess a range of health related issues and can be used irrespective of disease entity [7,12]. The WHOQOL-BREF is one such tool, which has been developed and validated across 20 countries in Africa, Asia and Latin America [12–16]. A hospital-based study in India used the WHOQOL-BREF to compare the QoL of 60 “trachomatous entropion” patients with age, sex and socio-economic status matched hospital patients without entropion or trichiasis [5]. However, about two-thirds of the cases had entropion without trichiasis, which precludes drawing conclusions about the QoL of trichiasis patients and the controls were not necessarily representative of the population.

Relatively little is known about the impact that trichiasis has on the lives of affected people. In this case-control study we measured the impact on vision and health-related QoL in Ethiopia, using standard WHO quantitative tools.

## Methods

### Ethics Statement

This study was reviewed and approved by the Food, Medicine and Healthcare Administration and Control Authority of Ethiopia, the National Health Research Ethics Review Committee of the Ethiopian Ministry of Science and Technology, Amhara Regional Health Bureau Research Ethics Review Board Committee, the London School of Hygiene and Tropical Medicine (LSHTM) Ethics Committee, and Emory University Institutional Review Board. Written informed consent in Amharic was obtained prior to enrolment from participants. If the participant was unable to read and write, the information sheet and consent form were read to them and their consent recorded by thumbprint.

### Study design and Participants

This case-control study was nested within a clinical trial of two alternative surgical treatments for trichiasis. For the trial 1000 trichiasis cases were recruited, and these were also enrolled into this QoL study. Cases were defined as individuals with one or more eyelashes touching the eyeball or with evidence of epilation in either or both eyes in association with tarsal conjunctival scarring. People with trichiasis from other causes, recurrent trichiasis and those <18 years of age were excluded. Trichiasis cases were identified mainly through community-based screening. Trichiasis screeners and counsellors (Eye Ambassadors) visited every household in their target village, identified and referred trichiasis cases to health facilities where surgical services were provided. Some cases self-presented or were referred by local health workers. Recruitment was done mainly from three districts of West Gojam Zone, Amhara Region, Ethiopia between February and May 2014. This area has one of the highest burdens of trachoma worldwide [17].

We recruited 200 matched controls to every fifth consecutive trichiasis case. Controls were individuals without clinical evidence or a history of trichiasis (including epilation), and who came from households without a family member with trichiasis or a history of trichiasis. Controls were individually matched with every fifth trichiasis case by location, sex and age (+/- two years). The research team visited the sub-village (30–50 households) of the trichiasis case that required a matched control. A list of all potentially eligible people living in the sub-village of the case was compiled with the help of the sub-village administrator. One person was randomly selected from this list using a lottery method, given details of the study and invited to participate if eligible. If a selected individual refused or was ineligible, another was randomly selected from the list. When eligible controls were not identified within the sub-village of the index case, recruitment was done in the nearest neighbouring sub-village, using the same procedures.

### Quality of life instruments

#### VRQoL

The WHO/PBD-VF20 was used to assess VRQoL [8]. It contains 20 questions sub-divided into three subscales: visual symptom, general functioning and psychosocial. Each question has a 5-point response option: one indicates the highest and five the lowest score. The first two questions measure the eyesight and amount of pain or discomfort the person is experiencing. The psychosocial questions assess the frequency of experiencing a specific vision-related problem, while the remaining items measure the difficulty associated with overall performance. Two translators translated the tool into Amharic independently. The two translations were compared and differences were discussed and resolved to develop a single, final version.

#### HRQoL

The WHOQOL-BREF was used to assess HRQoL [13]. It contains 26 questions, which assess QoL across four domains: physical health, psychological, social relationships and environment in the past four weeks [6,12,13]. The first two questions assess general QoL and health. Each item is scored on a 5-point scale. The Amharic version used in this study was provided by the WHO and has been previously validated and used in Ethiopia [18–20].

### Data collection

The VRQoL and HRQoL were administered orally by six trained Amharic speaking interviewers, because of the low literacy rate amongst participants. Data from trichiasis cases were collected at health facilities at the time of enrolment into the clinical trial, prior to surgery. Data from the controls were collected at their homes.

Data were also collected on general health problems and self-rated socioeconomic status (SES). For the self-rated socio-economic status, participants were asked to rate the wealth of their households in relation to other households in their village by choosing one of the following options: (1) very poor, (2) poor, (3) average, (4) wealthy or (5) very wealthy. In addition, data were collected on social relations, marriage and sleeping, through semi-structured questions including: “Do you feel ashamed or embarrassed due to the trichiasis?”, “Do you worry that you may lose your remaining eyesight due to the trichiasis?”, “Do you have a sleeping problem?” and “If yes, do you think your sleeping problem is related with the trichiasis?”

Presenting LogMAR (Logarithm of the Minimum Angle of Resolution) visual acuity at two metres was measured using “PeekAcuity” software on a Smartphone in a dark room [21]. We assessed contrast sensitivity with a prototype smartphone based test that presents the individual with calibrated grey scale spots against a white background, which they have to identify by touching the screen (www.peekvision.org). Unlike visual acuity, which is measured with high contrast, contrast sensitivity perhaps more accurately reflects the person’s everyday visual experience in varying conditions. Patients with normal visual acuity may have profound contrast sensitivity impairment. Therefore it is useful to measure contrast sensitivity while investigating VRQoL, as impairment could lead to decreased functioning and quality of life [22]. The ophthalmic examination was conducted using a 2.5x binocular magnifying loupe and a bright torch. Clinical signs were graded using the Detailed WHO FPC Grading System [23].

### Sample size

The sample size was calculated with the aim of detecting a three point difference in mean QoL score between trichiasis cases and controls [5]. The sample size of 1000 cases and 200 controls has 90% power to detect even minimal effect of trichiasis on QoL with an effect size of about 0.27 (effect size = QoL score difference (3)/SD (11)) with a Type I error of 5%.

### Analysis

Data were double-entered into Access (Microsoft), cleaned in Epidata 3.1 and transferred to Stata 11 (StataCorp) for analysis. Data were analysed as follows:

#### VRQoL

All items were grouped and scores added into their respective subscales: “General Vision” subscale (1 question); “Visual Symptoms” subscale (3 questions); “General Functioning” subscale (12 questions); and “Psychosocial” subscale (4 questions) [8]. The subscale scores were then converted into a scaled value out of one hundred, using the formula: ([individual score—lowest possible score]/[highest possible score—lowest possible score]) X100. Therefore, the person with the lowest possible VRQoL score would receive a scaled value of “0” and the person with the highest possible VRQoL score receives a scaled value of “100” [11].

#### HRQoL

Data were analysed following the WHOQOL protocol [6,13]. Three negatively framed items were reversed into a positive frame so higher scores denote higher QoL. To generate domain scores, questions were grouped into their respective domains and their scores totalled. The mean score of all items included in the domain was calculated and then multiplied by four. These scores then transformed to a 0 to 100 scale with the formula specified in the manual to allow comparison between domains made of unequal number of items [6].

#### Clinical data

The presenting visual acuity in the better eye was used in this analysis. For visual acuities of counting fingers or less, LogMAR values were attributed as follows: counting fingers, 2.0; hand movements, 2.5; perception of light, 3.0; no perception of light, 3.5 [24]. The LogMAR visual acuity scores were then categorised based on the WHO classifications: Normal vision, ≥6/18; moderate visual impairment, <6/18–≥6/60; severe visual impairment, <6/60–≥3/60; and blind, <3/60. Contrast sensitivity scores were grouped into quartiles. Corneal opacity and trichiasis grading in the more affected eye was used to test the association of these with QoL in trichiasis cases. Based on their trichiasis severity, cases were categorised into Minor Trichiasis (<6 lashes or evidence of epilation in <1/3^rd^ of the lash margin) and Major Trichiasis (≥6 lashes or evidence of epilation in ≥1/3^rd^ of the lash margin). Corneal opacity grading was categorised as either (i) no opacity/peripheral opacity or (ii) opacity involving the visual axis.

#### Comparison of cases and controls

All comparisons of cases and controls were adjusted for the matching variables: age and sex. The VRQoL analysis was also adjusted for socio-economic status and the HRQoL analysis adjusted for both socio-economic status and presence of health problems during the previous four weeks, as these factors may confound the association between trachomatous trichiasis and QoL. Logistic, linear and ordinal logistic regression methods were used for binary, continuous and ordered categorical outcome variable analysis, respectively. Linear regression models and the t-test were employed to compare significant differences in QoL scores and to generate mean and mean differences between cases and controls in each QoL subscale and domain, respectively.

#### Factors associated with QoL among cases

Linear regression was used to investigate the relationship between various factors with each VRQoL and HRQoL domain score (continuous variable) in trichiasis cases in a univariable and multivariable analysis. Tests for trend were undertaken in case of ordered categorical independent variables and significance was assessed using p-value for trend. Likelihood ratio-tests were employed to obtain p-values in categorical exposure variables. Variables that were associated with the outcome on univariable analyses at a level of p<0.05 were included in the multivariable analysis and then those with p<0.2 were retained in the model. To adjust for multiple comparisons, we used the Benjamini and Hochberg method, assuming a false discovery rate (FDR) of 5% [25].

#### Psychometric property evaluation

Construct validity of the VRQoL data was assessed through known-group difference and convergence validity using a linear regression model. In the HRQoL data discriminant (to distinguish differences between cases and controls) and construct validity were assessed using linear regression and Pearson’s correlation respectively. Cronbach’s alpha was used to test for internal consistency and reliability of the VRQoL and HRQoL data.

## Results

### Demographic and clinical characteristics of participants

Cases and controls were adequately matched for age (Table 1) and for geographical distribution across the 20 administrative units where recruitment was conducted. There were proportionately slightly more females among the 200 controls (83%) than the 1000 cases (76%). This occurred by chance as the 200 trichiasis cases used to determine the control matching characteristics had proportionately more females than in the full group of 1000 trichiasis cases. The trichiasis cases were more likely to be widowed or divorced, be from poorer households, and report other health problem in the past month than controls. For the analysis of socio-economic data we combined the “very poor” with the “poor” group and the “very wealthy” with “wealthy” group because of small numbers at both ends of the SES distribution. Trichiasis cases had substantially lower visual acuity scores (median LogMAR, 0.35; IQR, 0.15 to 0.6) than the controls (median LogMAR, -0.05; IQR, -0.1 to 0.1; Wilcoxon ranksum test, p<0.0001). Among trichiasis cases vision in the better eye was 6/18 or better in 63%, and 50% had minor trichiasis. The median duration of trichiasis among the cases was 5 years (IQR, 2–10).

**Table 1:** Demographic and clinical characteristics of participants.

Variables	Cases		Controls		p-value
	n / 1000	(%)	n / 200	(%)	
**Age, mean (SD)**	47.3 years	(13.5)	45.9 years	(13.3)	-
**Gender, female**	765	(76.5)	167	(83.6)	-
**Illiterate**	886	(88.6)	170	(85.0)	0.005
**Marital status**					
Married	646	(64.6)	162	(81.0)	0.0001^‡^
Widowed	203	(20.3)	27	(13.5)	
Divorced	119	(11.9)	9	(4.5)	
Single	32	(3.2)	2	(1.0)	
**Job** ^‡^					
Farmer	839	(83.9)	168	(84.0)	0.006^‡^
Employed/self employed	52	(5.2)	17	(8.5)	
Daily labourer	46	(4.6)	4	(2.0)	
No job	63	(6.3)	11	(5.5)	
**Self rated wealth** *					
Very wealthy/ Wealthy	32	(3.2)	30	(15.0)	<0.0001^†^
Middle	447	(44.7)	135	(67.5)	
Very Poor / Poor	521	(52.1)	35	(17.5)	
**Health problem**					
No	628	(62.8)	172	(86.0)	<0.0001
Yes	372	(37.2)	28	(14.0)	
**Visual Acuity—better eye**					
Normal (≥6/18)	638	(63.8)	194	(97.0)	<0.0001^†^
Moderate visual impairment (<6/18 to ≥6/60)	327	(32.7)	4	(2.0)	
Severe visual impairment (<6/60 to ≥3/60)	18	(1.8)	1	(0.5)	
Blind (<3/60)	17	(1.7)	1	(0.5)	
**Contrast sensitivity**					
1 (Best)	336	(33.6)	142	(71.0)	<0.0001^†^
2	193	(19.3)	33	(16.5)	
3	249	(24.9)	17	(8.5)	
4 (Worst)	222	(22.2)	8	(4.0)	

p-values are calculated using logistic regression and adjusted for age and gender, with the exception for age, which was calculated using linear regression.

^***‡***^ Combined p-value from likelihood ratio-test.

^†^ P-value for trend.

* We merged “very wealthy” and “wealthy” and “very poor” and “poor” because of small numbers at the extremes of the distribution, to create three levels of socio-economic status measure to facilitate data modelling.

### Vision related quality of life

The trichiasis cases had substantially lower VRQoL scores than the controls in all four subscales (p<0.0001) (Table 2). The largest differences between cases and controls were found for visual symptoms (mean difference, 51.5; 95%CI, 48.5–54.5) and the smallest in general functioning (mean difference, 24.1; 95%CI, 21.1–27.1). In a sub-group analysis, trichiasis cases with normal vision had significantly lower VRQoL in all subscales than controls with normal vision (Table 3).

**Table 2:** Comparison of mean domain scores of VRQoL and HRQoL of trichiasis cases and controls.

Domain	TT Cases		Controls		Mean difference		p-value
	Mean	(95% CI)	Mean	(95% CI)	Mean	(95% CI)	
***VRQoL*** ^a^							
Overall eyesight	45.9	(44.5–47.3)	95.4	(93.2–97.5)	49.5	(46.3–52.7)	<0.0001
Visual symptom	46.0	(44.7–47.3)	97.5	(96.1–98.9)	51.5	(48.5–54.5)	<0.0001
General Functioning	73.7	(72.4–75.0)	97.7	(96.4–99.1)	24.1	(21.1–27.1)	<0.0001
Psychosocial	69.1	(67.4–70.7)	98.1	(96.8–99.5)	29.1	(25.3–32.8)	<0.0001
***HRQoL*** ^b^							
***General Facet Items***							
Overall quality of life	34.5	(33.3–35.7)	64.6	(61.9–67.3)	30.1	(27.2–33.1)	<0.0001
Overall health	38.2	(37.0–39.5)	71.7	(69.5–74.0)	33.5	(30.4–36.5)	<0.0001
***Domains***							
Physical health	47.4	(46.4–48.3)	79.8	(77.9–81.7)	32.4	(30.1–34.7)	<0.0001
Psychological	58.7	(58.0–59.5)	80.5	(79.0–82.0)	21.8	(20.0–23.7)	<0.0001
Social	51.7	(50.3–53.1)	72.1	(69.7–74.5)	20.4	(17.1–23.7)	<0.0001
Environment	38.7	(38.0–39.4)	62.0	(60.4–63.6)	23.3	(21.6–24.9)	<0.0001

^**a**^ p-values were calculated by linear regression and adjusted for age, gender and self-rated wealth.

VRQoL = Vision Related Quality of Life

^**b**^ p-values were calculated by linear regression and adjusted for age, gender, self-rated wealth and other health problem in the past month

HRQoL = Health Related Quality of Life

**Table 3:** Comparison of mean VRQoL and HRQoL scores of trichiasis cases and controls with normal vision (presenting visual acuity of ≥6/18 in the better eye).

Domain	TT Cases With Normal Vision n = 638		Controls With Normal Vision n = 194		Mean difference		p-value
	Mean	(95% CI)	Mean	(95% CI)	Mean	(95% CI)	
***VRQoL*** ^a^							
Overall eyesight	50.5	(48.8–52.2)	96.9	(95.2–98.6)	46.4	(43.2–49.6)	<0.0001
Visual symptom	49.4	(47.8–51.0)	98.5	(97.5–99.5)	49.0	(46.1–52.0)	<0.0001
General Functioning	79.8	(78.5–81.2)	98.7	(97.8–99.6)	18.9	(16.4–21.4)	<0.0001
Psychosocial	74.1	(72.2–76.1)	98.8	(97.7–99.9)	24.7	(21.1–28.2)	<0.0001
***HRQoL*** ^b^							
***General Facet Items***							
Overall quality of life	37.0	(35.5–38.5)	64.9	(62.2–67.7)	28.0	(24.9–31.0)	<0.0001
Overall health	41.7	(40.2–43.3)	72.2	(70.0–74.3)	30.4	(27.4–33.5)	<0.0001
***Domains***							
Physical health	51.5	(50.4–52.6)	80.3	(78.5–82.1)	28.9	(26.5–31.0)	<0.0001
Psychological	60.2	(59.2–61.2)	80.9	(79.4–82.3)	20.7	(18.8–22.6)	<0.0001
Social	54.9	(53.2–56.7)	72.5	(70.1–74.9)	17.5	(14.1–20.9)	<0.0001
Environment	39.9	(39.1–40.9)	62.3	(60.7–63.8)	22.3	(20.6–24.1)	<0.0001

^**a**^ p-values were calculated by linear regression and adjusted for age, gender and self-rated wealth.

VRQoL = Vision Related Quality of Life

^**b**^ p-values were calculated by linear regression and adjusted for age, gender, self-rated wealth and other health problem in the past month

HRQoL = Health Related Quality of Life

The relationship between VRQoL and various demographic and clinical characteristics among individuals with trichiasis are presented in Table 4. VRQoL scores were lower in all domains with increasing age, severity and duration of trichiasis and with decreasing visual acuity and contrast sensitivity scores. Scores were also lower in females, illiterate individuals and in cases with central corneal opacity.

**Table 4:** Univariable^a^ and Multivariable^b^ associations of VRQoL with demographic and clinical characteristics among trichiasis cases.

Variable	Overall eyesight		Visual symptom		General functioning		Psychosocial	
	*Mean*	*(95% CI)*	*Mean*	*(95% CI)*	*Mean*	*(95% CI)*	*Mean*	*(95% CI)*
**Age Groups (years)**								
≤ 29	55.1	(50.8–59.4)	52.5	(48.7–56.4)	83.7	(80.0–87.3)	74.4	(69.4–79.3)
30–39	51.3	(48.2–54.4)	48.2	(45.6–50.9)	81.5	(79.3–83.7)	74.2	(70.7–77.8)
40–49	46.6	(43.9–49.4)	47.1	(44.3–49.8)	75.7	(73.4–78.0)	69.2	(65.7–72.6
50–59	43.0	(40.2–45.8)	42.7	(40.0–45.4)	71.2	(68.4–73.9)	66.8	(63.3–70.3)
60–69	42.5	(39.1–45.9)	46.0	(42.6–49.4)	68.5	(65.0–71.9)	68.7	(64.5–72.9)
≥ 70	32.2	(30.7–39.8)	39.1	(34.6–43.6)	56.8	(51.4–62.1)	58.8	(53.0–64.6)
p-value ^a^ ^†^	<0.0001		<0.0001		<0.0001		<0.0001	
p-value ^b^ ^†^	-		0.069		0.010		0.0048	
**Gender**								
Male	48.2	(45.2–51.2)	49.1	(46.6–51.7)	77.8	(75.2–80.4)	72.7	(69.5–76.0)
Female	45.2	(43.6–46.7)	45.0	(43.5–46.5)	72.4	(70.9–73.9)	67.9	(66.0–69.9)
p-value ^a^	0.07		0.008		0.0007		0.02	
p-value ^b^	-		-		0.034		-	
**Literacy**								
Literate	52.6	(48.6–56.7)	52.3	(48.8–55.7)	84.2	(81.3–87.20	77.2	(73.1–81.4)
Illiterate	45.0	(43.5–46.5)	45.2	(43.8–46.6)	72.3	(70.9–73.7)	68.0	(66.2–69.8)
p-value ^a^	0.0006		0.0006		<0.0001		0.0005	
p-value ^b^	0.19		-		0.049		-	
**Trichiasis severity**								
Minor trichiasis (<6 lashes)	47.7	(45.8–49.6)	48.4	(46.5–50.2)	75.8	(74.0–77.6)	71.1	(68.8–73.4)
Major trichiasis (≥6 lashes)	44.0	(42.1–46.0)	43.6	(41.8–45.4)	71.6	(69.6–73.5)	67.0	(64.7–69.4)
p-value ^a^	0.009		0.0003		0.002		0.02	
p-value ^b^	-		-		-		-	
**Trichiasis duration in years**								
<2	53.8	(50.5–57.1)	57.4	(54.5–60.3)	81.5	(78.4–84.7)	77.7	(73.8–81.5)
2–4	46.9	(44.4–49.4)	46.5	(44.2–48.8)	74.9	(72.6–77.1)	68.8	(65.8–71.8)
5–9	44.9	(42.1–47.7)	44.3	(41.7–46.9)	73.5	(70.8–76.1)	68.7	(65.4–72.0)
10–15	42.0	(38.2–45.7)	40.7	(37.3–44.1)	70.6	(67.4–73.9)	65.0	(60.5–69.4)
≥15	40.4	(36.9–43.9)	40.0	(66.7–43.3)	66.1	(62.4–69.9)	64.4	(60.0–68.7
p-value ^a^ ^†^	*<0*.*0001*		*<0*.*0001*		*<0*.*0001*		*<0*.*0001*	
p-value ^b^ ^†^	<0.0001		<0.0001		<0.0001		0.0004	
**Corneal opacity**								
No/peripheral opacity	50.2	(48.4–52.0)	49.2	(47.5–50.9)	78.0	(76.4–79.5)	73.9	(71.8–76.0)
Opacity involving the visual axis	40.4	(38.4–42.4)	41.9	(40.0–43.8)	68.3	(66.1–70.4)	62.9	(60.4–65.5)
p-value ^a^	*<0*.*0001*		*<0*.*0001*		*<0*.*0001*		*<0*.*0001*	
p-value ^b^	0.0002		0.0023		0.0077		0.0002	
**Presenting VA in the better eye**								
Normal (≥6/18)	50.5	(48.8–52.2)	49.4	(47.8–51.0)	79.8	(78.5–81.2)	74.1	(72.2–76.1)
Moderate visual impairment (<6/18 to ≥6/60)	39.7	(37.5–41.8)	40.9	(38.8–43.1)	65.3	(62.9–67.6)	62.3	(59.5–65.2)
Severe visual impairment (<6/60 to ≥3/60)	23.6	(15.7–31.6)	35.6	(26.0–45.3)	46.1	(36.0–56.1)	45.1	(32.7–57.6)
Blind (<3/60)	14.7	(5.6–23.9)	26.0	(16.5–35.4)	33.7	(18.9–48.7)	33.8	(18.3–49.4)
p-value ^a^ ^†^	<0.0001		<0.0001		<0.0001		<0.0001	
p-value ^b^ ^†^	<0.0001		0.0002		<0.0001		<0.0001	
**Contrast sensitivity**								
4 (Best score)	54.2	(51.8–56.6	52.1	(49.9–54.2)	83.6	(81.9–85.3)	78.3	(75.8–80.7)
3	46.8	(43.8–49.8)	46.8	(43.9–49.7)	75.4	(72.9–77.8)	70.0	(66.3–73.7)
2	42.6	(40.1–45.0)	44.2	(41.6–46.8)	71.9	(69.6–74.2)	67.0	(63.9–70.1)
1 (Worst score)	36.1	(33.4–38.9)	38.1	(35.5–40.7)	59.1	(55.8–72.4)	56.6	(52.9–60.4)
p-value ^a^ ^†^	<0.0001		<0.0001		<0.0001		<0.0001	
p-value ^b^ ^†^	<0.0001		<0.0001		<0.0001		<0.0001	
**Self rated wealth**							
Very wealthy / Wealthy	53.9	(45.3–62.5)	47.1	(38.9–55.4)	83.5	(76.4–90.7)	80.3	(72.3–88.3)
Middle	48.8	(46.8–50.8)	48.2	(46.4–50.1)	78.3	(76.6–79.9)	72.7	(70.4–75.1)
Poor / Very Poor	42.8	(40.9–44.8)	44.0	(42.2–45.8)	69.1	(67.2–71.1)	65.2	(62.9–67.6)
p-value ^a^ ^†^	<0.0001		0.0037		<0.0001		<0.0001	
p-value ^b^ ^†^	0.029		-		0.0001		0.0019	

^a^ P-values from univariable analysis.

^b^ P-values from multivariable analysis. All p-values are calculated using linear regression. For ordinal exposures with three or more categories the

^†^p-values are calculated for trend. Using the Benjamini and Hochberg method, only tests with a p-value below 0.034 have a False Discovery Rate of <5%. Variables with univariable p<0.05 were included in the multivariable model, then those with p>0.2 were excluded (dashed line) from the final model after likelihood ratio- test. VRQoL = Vision related Quality of Life

In multivariable analysis, lower overall VRQoL scores were found in those with longer trichiasis duration, central corneal opacity, visual impairment and poor contrast sensitivity (Table 4). In addition to these, older age and female gender were associated with lower VRQoL score in the general functioning subscale. In the model, people with Major Trichiasis had lower VRQoL score in the visual symptom domain after controlling for potential confounders (p = 0.011), however, this is confounded by duration of trichiasis and was therefore dropped from the final model (Table 4).

### Health related quality of life

The trichiasis cases had substantially lower overall HRQoL (p<0.0001) and overall self rated health (p<0.0001) scores than the controls (Table 2). Strikingly, 55.4% of trichiasis cases rated their overall QoL “poor” or “very poor” compared to only 9.5% of controls (p<0.0001) and 5.6% of cases rated their overall QoL “good” or “very good” compared to 59.5% of controls (p<0.0001). Across all four domains there were substantial differences in the QoL scores of cases and controls (all p<0.0001, Table 2). The largest difference was seen in the physical health domain (mean difference, 32.4; 95%CI, and 30.1–34.7). Trichiasis cases with normal vision had significantly lower HRQoL in all subscales than controls with normal vision (Table 3).

Among trichiasis cases, overall HRQoL and the four domain scores decreased with increasing age (except for environment domain), decreasing self-rated wealth, visual acuity and contrast sensitivity scores (Table 5). They were also lower in divorced/widowed, illiterate individuals, females and those with other health problems in the past month. Daily labourers, the unemployed and those with central corneal opacity had lower overall QoL and domain scores except for the environment domain. Participants with longer duration trichiasis had lower physical, psychological and social domain scores.

**Table 5:** Univariable^a^ and Multivariable^b^ associations of HRQoL with demographic and clinical characteristics among trichiasis cases.

Variable	Overall quality of life		Physical domain		Psychological domain		Social domain		Environmental domain	
	Mean	(95% CI)	Mean	(95% CI)	Mean	(95% CI)	Mean	(95% CI)	Mean	(95% CI)
**Age Groups (years)**										
≤ 29	38.4	(34.8–42.0)	60.7	(58.2–63.3)	63.4	(61.1–65.6)	61.0	(56.8–65.1)	41.1	(38.9–43.3)
30–39	37.7	(35.0–40.4)	54.3	(52.5–56.1)	60.6	(58.8–62.4)	58.9	(55.9–62.0)	41.5	(39.9–43.0)
40–49	37.9	(35.5–40.2)	49.1	(47.4–50.9)	58.9	(57.4–60.4)	56.6	(53.8–59.4)	38.8	(37.4–40.1)
50–59	32.6	(29.9–35.3)	44.2	(42.3–46.1)	57.6	(56.0–59.2)	46.3	(43.1–49.4)	38.2	(36.7–39.6)
60–69	30.9	(28.0–33.8)	40.7	(36.6–42.8)	56.8	(54.7–58.8)	42.1	(39.0–45.3)	36.4	(34.8–38.0)
≥ 70	26.0	(25.0–30.0)	32.7	(29.8–35.6)	55.4	(52.8–57.9)	42.8	(39.1–46.6)	35.3	(33.2–37.4)
p-value ^a^ ^†^	<0.0001		<0.0001		<0.0001		<0.0001		<0.0001	
p-value ^b^ ^†^	-		<0.0001		0.13		<0.0001		0.048	
**Gender**										
Male	36.1	(33.47–38.4)	51.9	(50.0–53.9)	60.1	(58.6–61.5)	57.7	(54.9–60.5)	41.0	(39.6–42.3)
Female	34.0	(32.6–35.4)	46.0	(44.9–47.1)	58.3	(57.4–59.2)	49.8	(48.2–51.4)	38.0	(37.2–38.8)
p-value ^a^	0.16		<0.0001		0.06		<0.0001		<0.0001	
p-value ^b^	-		<0.0001		-		-		0.024	
**Literacy**									
Literate	39.9	(36.5–43.3)	56.4	(53.7–59.1)	61.4	(59.1–63.7)	59.3	(55.2–63.3)	42.5	(40.3–44.7)
Illiterate	33.8	(32.5–35.1)	46.2	(45.2–47.2)	58.4	(57.6–59.2)	50.7	(49.2–52.2)	38.2	(37.5–38.9)
p-value ^a^	0.0016		<0.0001		0.02		0.0001		<0.0001	
p-value ^b^	-		-		-		-		0.19	
**Marital status**										
Married	39.4	(38.1–40.8)	51.0	(49.9–52.1)	60.9	(60.0–61.7)	60.6	(58.9–62.2)	40.4	(39.6–41.2)
Single	32.0	(23.4–40.7)	53.6	(46.5–60.7)	55.9	(50.1–61.6)	41.4	(34.9–47.9)	37.6	(32.5–42.7)
Divorced/widowed	24.8	(22.7–20.7)	39.4	(37.8–41.1)	54.7	(53.2–56.2)	34.9	(33.2–36.6)	35.4	(34.3–36.5)
p-value ^a^										
Married vs. Single	0.03		0.33		0.02		<0.0001		0.14	
Married vs. Divorced/widowed	<0.0001		<0.0001		<0.0001		<0.0001		<0.0001	
P value ^b^										
Married vs. Single	0.023		0.35		0.023		<0.0001		0.0043	
Married vs. Divorced/widowed	<0.0001		0.18		0.0076		<0.0001		0.77	
**Job**										
Farmer	36.3	(35.0–37.6)	48.0	(47.0–49.0)	59.5	(58.8–60.3)	53.1	(51.6–54.7)	38.9	(38.2–39.6)
Employed/self-employed	30.3	(24.5–36.0)	51.2	(46.9–55.5	56.9	(53.1–60.7)	45.2	(39.5–50.9)	38.7	(35.8–41.5)
Daily labour	17.9	(12.8–23.0)	43.7	(38.5–48.9)	52.2	(46.3–58.0)	42.6	(35.6–49.6)	37.4	(33.4–41.3)
No job (including students)	26.2	(20.4–32.0)	38.4	(33.4–43.3)	54.6	(50.4–58.9	44.4	(39.0–49.8)	36.7	(33.3–40.1)
p-value ^a^										
Farmer vs. Employed/self-employed	0.03		0.14		0.13		0.01		0.89	
Farmer vs. Daily labour	<0.0001		0.06		0.0001		0.002		0.34	
Farmer vs. No job	<0.0001		<0.0001		0.002		0.003		0.12	
p-value ^b^										
Farmer vs. Employed/self-employed	0.55		0.030		0.62		-		-	
Farmer vs. Daily labour	<0.0001		0.17		0.0086		-		-	
Farmer vs. No job	0.037		0.0005		0.18		-		-	
**Trichiasis severity**									
Minor trichiasis (<6 lashes)	35.2	(33.6–36.9)	48.7	(47.4–50.1)	58.9	(57.9–60.0)	53.7	(51.7–55.7)	39.4	(38.5–40.3)
Major trichiasis (≥6 lashes)	33.7	(32.0–35.5)	46.0	(44.6–47.4)	58.3	(57.4–59.7)	49.6	(47.6–51.6)	38.0	(37.0–39.0)
p-value ^a^	0.22		0.005		0.60		0.004		0.05	
p-value ^b^	-		-		-		-		-	
**Trichiasis duration**									
<2	35.0	(32.2–37.8)	50.2	(47.8–52.6)	59.4	(57.6–61.3)	51.4	(47.8–55.0)	39.7	(38.1–41.2)
2–4	34.7	(32.4–37.0)	49.3	(47.6–51.0)	60.1	(58.7–61.4)	54.9	(52.1–57.6)	37.9	(36.6–39.1)
5–9	35.9	(33.5–38.4)	47.6	(45.7–49.5)	58.2	(56.5–59.8)	51.7	(47.8–54.5)	40.0	(38.6–41.4)
10–15	34.7	(31.3–38.1)	46.1	(43.7–48.5)	59.6	(57.5–61.6)	53.4	(49.9–56.9)	39.3	(37.6–41.0)
≥15	31.2	(28.2–34.2)	41.7	(39.1–44.3)	55.8	(53.7–57.9)	45.0	(41.7–48.3)	36.7	(35.0–38.4)
p-value ^a^ ^†^	0.11		<0.0001		0.005		0.003		0.14	
p-value ^b^ ^†^	-		-		-		-		-	
**Corneal Opacity**										
No/peripheral opacity	36.6	(35.0–38.1)	50.3	(49.0–51.5)	59.8	(58.8–60.8)	53.4	(51.5–55.3)	39.3	(38.4–40.2)
Opacity involving the visual axis	31.9	(30.1–33.7)	43.7	(42.3–45.1)	57.4	(56.2–58.6)	49.5	(47.4–51.5)	37.9	(36.9–38.9)
p-value ^a^	0.0002		<0.0001		0.003		0.006		0.04	
p-value ^b^	-		0.057		-		-		-	
**Presenting VA in the better eye**									
Normal (≥6/18)	37.0	(33.5–38.4)	51.5	(50.4–52.6)	60.2	(59.2–61.2)	54.9	(53.2–56.7)	39.9	(39.1–40.8)
MVI (<6/18 to ≥6/60)	31.0	(28.9–33.2)	40.8	(39.2–42.4)	56.6	(55.4–57.9)	46.0	(43.6–48.4)	37.0	(35.8–38.1)
SVI (<6/60 to ≥3/60)	23.6	(13.6–33.6)	34.9	(29.0–40.9)	54.9	(48.1–61.6)	46.8	(34.7–58.8)	33.0	(27.6–38.3)
Blind (<3/60)	19.1	(8.4–29.8)	31.1	(21.5–40.7)	49.0	(39.2–58.8)	43.1	(30.3–56.0)	31.4	(24.3–38.5)
p-value ^a^ ^†^	<0.0001		<0.0001		<0.0001		<0.0001		<0.0001	
p-value ^b^ ^†^	0.068		0.0010		0.054		-		0.17	
**Contrast sensitivity**										
1 (Best score)	36.5	(36.4–40.5)	55.5	(54.0–57.0)	61.5	(60.2–62.7)	58.1	(55.6–60.4)	41.0	(39.8–42.2)
2	36.9	(34.2–39.6)	46.6	(44.6–48.7)	58.2	(56.5–59.9)	51.9	(48.6–55.2)	40.0	(38.5–41.3)
3	32.0	(29.7–34.4)	45.3	43.6–47.0)	58.5	(57.0–59.9)	49.3	(46.4–52.1)	38.1	(36.9–39.3)
4 (Worst score)	29.2	(26.6–31.7)	38.0	(36.1–39.9)	55.3	(53.5–57.2)	44.5	(41.6–47.4)	34.9	(33.5–36.3)
p-value ^a^ ^†^	<0.0001		<0.0001		<0.0001		<0.0001		<0.0001	
p-value ^b^ ^†^	-		0.0008		0.15		0.18		0.027	
**Other health problems**										
No	37.2	(35.8–38.6)	51.8	(50.6–52.9)	60.4	(59.5–61.4)	53.4	(51.6–55.2)	39.8	(39.0–40.7)
Yes	30.0	(27.9–32.1)	40.0	(38.5–41.4)	55.9	(54.6–57.1)	48.7	(46.6–50.9)	36.8	(35.8–37.8)
p-value ^a^	<0.0001		<0.0001		<0.0001		0.002		<0.0001	
p-value ^b^	0.0018		<0.0001		0.0002		-		0.080	
**Self rated wealth**										
Very wealthy / Wealthy	54.7	(48.4–60.9)	56.8	(52.0–61.6)	62.8	(57.8–67.7)	59.4	(50.6–68.2)	46.3	(42.4–50.2)
Middle	46.0	(44.6–47.3)	52.4	(51.0–53.7)	62.4	(61.4–63.4)	59.8	(57.9–61.7)	42.7	(41.7–43.6)
Poor / Very Poor	23.4	(22.0–24.8)	42.5	(41.2–43.8)	55.4	(54.3–56.4)	44.2	(42.3–46.1)	34.8	(34.0–35.7)
p-value ^a^ ^†^	<0.0001		<0.0001		<0.0001		<0.0001		<0.0001	
p-value ^b^ ^†^	<0.0001		<0.0001		<0.0001		<0.0001		<0.0001	

^a^ P-values from univariable analysis.

^b^ P-values from multivariable analysis. P-values are calculated using linear regression. For ordinal exposures with three or more categories the

^†^p-values are calculated for trend. Using the Benjamini and Hochberg method, only tests with a p-value below 0.03 have a False Discovery Rate of <5%. Variables with univariable p<0.05 were included in the multivariable model, then those with p>0.2 were excluded (dashed line) from the final model after likelihood ratio test. MV = Moderate Visual Impairment; SVI = Severe Visual Impairment. HRQoL = Health related Quality of Life.

Multivariable analyses identified predictors of HRQoL among trichiasis cases (Table 5). Lower self-rated wealth was associated with lower QoL scores in all domains. Poorer overall QoL was related to not having a marriage partner, visual impairment, being a daily labourer and presence of other health problems. Older participants, females, the unemployed, those with visual impairment, poor contrast sensitivity score and other health problems were associated with lower physical domain scores. Daily labouring, not having a marriage partner and presence of other health problems were associated with lower psychological domain scores.

### Impact of trichiasis on daily life

Among the 200 controls, 198 (99%) reported no ocular pain or discomfort. In contrast, among the 1000 trichiasis cases, 143 (14.3%), 281 (28.1%) and 562 (56.2%) reported mild, moderate and severe ocular pain or discomfort, respectively (p<0.0001). The cases reported the following effects of trichiasis: 596 (59%) felt ashamed or embarrassed; 913 (91.3%) worried that they might lose their remaining eyesight; 70 (7.0%) had been troubled in their marriage and ignored by their marriage partner; 681 (68.1%) reported sleeping problems, largely due to pain (675/681 (67.5%)) from the trichiasis.

### Validity and reliability of the QoL Data

Satisfying the known-groups difference criteria, the trichiasis cases had significantly lower VRQoL and HRQoL scores in all domains (p<0.0001) than the controls (Table 2). With respect to convergence validity, worsening visual acuity and contrast sensitivity scores, trichiasis duration and central corneal opacity were significantly associated with lower scores in all four domains of VRQoL (Table 4). The VRQoL data were reliable after being assessed for internal consistency with a Cronbach’s alpha: coefficients of >0.80. The overall QoL data showed very high internal consistency with a Cronbach’s alpha of 0.90. The physical health, psychological, social and environment domains demonstrated internal consistency with Cronbach’s alpha of 0.87, 0.65, 0.47, and 0.64, respectively.

## Discussion

Trachomatous trichiasis results in considerable morbidity even before the development of irreversible visual impairment or blindness from corneal opacification. The eyelashes constantly rub the cornea causing irritation and pain [26]. However, despite these important consequences there are surprisingly limited data on the impact of trichiasis on quality of life. Moreover, few studies have investigated the resulting functional physical impairment [27,28]. The psychological and social effects of trichiasis have usually been overlooked. Little information has previously been collected using validated tools. In response, this study was conducted to address these gaps, using standard WHO QoL instruments, and found that trachomatous trichiasis has a very profound impact on both VRQoL and HRQoL, even prior to the development of visual impairment.

### Vision related quality of life

Overall, the VRQoL of trichiasis cases was substantially lower in all domains compared to controls. When we restricted the analysis to people with a visual acuity of 6/18 or better, the difference in VRQoL was of a similar magnitude and was highly significant. This is an important observation, which demonstrates that trichiasis reduces VRQoL even before impairment of visual acuity develops.

We found that, of the four sub-scales, the one with the largest difference was the visual symptom subscale. This is a composite of questions about visual functioning, pain/discomfort, glare and light/dark adaption. The general functioning subscale showed the smallest difference compared to controls. This may be because approximately two-thirds of trichiasis cases had normal vision, and this subscale includes items on vision difficulties and role limitation.

Consistent with our study, several other studies have demonstrated the effect trichiasis has on physical functioning [26–28]. A population-based study in Tanzania found trichiasis without visual impairment results in limitation of physical functioning in women that was comparable to limitation associated with visual impairment from other causes [27]. A qualitative study of 23 women with trichiasis in Niger (without a control group for comparison) reported trichiasis had marked effects on the general well-being of these individuals and was linked to physical disability and inability to work and earn an income [26]. In a study conducted in southern Ethiopia, 61% of trichiasis cases reported difficulty in physical functioning including walking, recognizing faces and performing day-to-day farming activities [28].

Among trichiasis cases, VRQoL was significantly lower with central corneal opacity, increasing trichiasis duration and decreasing visual acuity and contrast sensitivity scores. This suggests that trichiasis cases had significantly lower contrast sensitivity than the controls. Other studies have found conditions such as dry eyes and reduced tear break-up time, resulting from progressive conjunctival scarring and ocular epithelial tissues damage, are associated with reduced contrast sensitivity score [29,30]. Impaired contrast sensitivity score can greatly affect the person’s ability to recognise objects and perform daily activities under different conditions. A recent study conducted on glaucoma patients revealed that contrast sensitivity plays a major role in daily functioning and VRQoL; and the contrast sensitivity score was correlated with VRQoL indicators such as facial recognition, finding objects, motion detection and general vision [22]. Poor contrast sensitivity has been associated with physical injuries from accidents, suggesting that it would greatly hamper overall well being [31,32]. The association between trichiasis severity and the visual symptom subscale was weakened after including trichiasis duration in the multivariable regression model. Severity and duration are not independent of each other. The general functioning subscale of the VRQoL and physical health domain in HRQoL were lower in females than males. Although the reason is not apparent, a similar finding has been reported in a Tanzanian study: women without visual impairment were more likely to have functional limitation than their male counterparts [27].

### Health related quality of life

Trichiasis cases had a poorer HRQoL than controls in all domains, again even without visual impairment. Strikingly, the physical domain in the WHOQOL-BREF, which includes questions on pain and discomfort, had the highest mean score difference between cases and controls, emphasising the suffering trichiasis causes. Among the QoL domains, the environmental domain had the lowest score in both trichiasis cases and controls. This domain is built from items such as satisfaction with financial resources, access to health service, transport, information and leisure activities, which generally have low availability in the communities where this study was conducted. Hence, participants would be anticipated to have a lower rate of satisfaction for these items.

The WHOQOL-BREF has been used to assess HRQoL of entropion patients (with and without trichiasis) [5]. Apart from the environment domain scores of the trichiasis cases, the average QoL scores of trichiasis cases and controls in all domains in the Indian study were generally lower than those we recorded in Ethiopia [5]. This difference could be attributed to two things. Firstly, perceptions towards QoL in Indian and Ethiopian communities could be different. Secondly, in the Indian study, all participants were recruited from hospital, compared to community-based recruitment in our study. Hospital participants might be more likely to report poor QoL than people in the community [14]. In contrast to trichiasis, there is an extensive literature about the impact of cataract on QoL [7,9–11,33]. However, there are fundamental differences between cataract and trichiasis in the nature of visual loss, pain and other symptoms they cause.

### Validity and reliability of QoL data

These tools have previously been reported to be valid and reliable in studies conducted in similar settings to this study [9–11,14,18–20]. In this study, both the VRQoL and HRQoL data measured what they were intended to measure (construct validity) by demonstrating significant differences in the scores between groups known to be different; cases and controls had lower and higher scores respectively. The VRQoL data also showed that sub-scales correlate well with measures of similar constructs (convergent validity) such as visual acuity and contras sensitivity where worsening in these measures is associated with lower VRQoL subscale scores. There was evidence of higher homogeneity among the items in each VRQoL subscale (internal consistency) than the generally accepted criteria of >0.70. In the HRQoL data, the overall QoL and the physical health domain items were internally consistent and reliable in measuring the same construct, while the psychological, social relations and environment domains had less internal consistency. Similar psychometric properties have been reported in the field trial results of this tool in other countries [14]. The lower alpha score for the social relationship domain is anticipated as its analysis is based on three items instead of the generally recommended minimum four for evaluating internal consistency [6]. Hence, this domain’s results should be interpreted in caution, as there was insufficient evidence that the items in this domain are always measuring the same construct.

### Strengths and limitations

This is a large case-control study examining the impact of trichiasis on QoL. We used tools validated in settings similar to this study setting. The VRQoL tool has been validated in Kenya while the HRQoL tool has been tested and used in other studies in Ethiopia. The study has some limitations. Perfect matching was not achieved in terms of gender, resulting in more females in the controls than the trichiasis cases. However, all comparisons between cases and controls were adjusted for gender, age and self-rated wealth. A community-based screening method was employed to identify trichiasis cases. Although we think that this was an efficient and comprehensive approach to finding cases, it is possible that some cases might have been missed, particularly those with mild disease who may be less likely to come forward, which could lead to an overestimation of the QoL scores for trichiasis cases in general. Trichiasis is strongly associated with a poorer QoL, However, the cross-sectional nature of this study precludes us from drawing definite conclusions about causality. This question is being investigated by reassessing this group of people one year after surgery.

### Conclusions

In this Ethiopian population, we found that trichiasis cases have significantly lower VRQoL and HRQoL than controls regardless of visual impairment. The results provide solid data for advocacy and encourage programme leaders and funders to secure resources to promote trichiasis intervention. Trichiasis inflicts considerable physical and psychosocial trauma including sleep disturbance, low self-esteem and possibly a less stable marriage. The burden of trichiasis goes beyond visual loss. Timely treatment is needed not only to prevent visual loss but also alleviate physical and psychological suffering and social exclusion of trichiasis patients, thereby improving their physical and psychological health, general functioning and social relations. The comprehensive SAFE strategy is needed to prevent the development of trichiasis. The long-term effect of trichiasis surgery on VRQoL and HRQoL in trichiasis patients needs to be measured in longitudinal studies.

## Supporting Information

S1 Checklist STROBE Checklist. (DOCX)
